# Inhibition of hERG Potassium Channels by Celecoxib and Its Mechanism

**DOI:** 10.1371/journal.pone.0026344

**Published:** 2011-10-24

**Authors:** Roman V. Frolov, Irina I. Ignatova, Satpal Singh

**Affiliations:** 1 Department of Pharmacology and Toxicology, State University of New York, Buffalo, New York, United States of America; 2 Division of Biophysics, Department of Physical Sciences, University of Oulu, Oulun Yliopisto, Finland; Hôpital Robert Debré, France

## Abstract

**Background:**

Celecoxib (Celebrex), a widely prescribed selective inhibitor of cyclooxygenase-2, can modulate ion channels independently of cyclooxygenase inhibition. Clinically relevant concentrations of celecoxib can affect ionic currents and alter functioning of neurons and myocytes. In particular, inhibition of Kv2.1 channels by celecoxib leads to arrhythmic beating of *Drosophila* heart and of rat heart cells in culture. However, the spectrum of ion channels involved in human cardiac excitability differs from that in animal models, including mammalian models, making it difficult to evaluate the relevance of these observations to humans. Our aim was to examine the effects of celecoxib on hERG and other human channels critically involved in regulating human cardiac rhythm, and to explore the mechanisms of any observed effect on the hERG channels.

**Methods and Results:**

Celecoxib inhibited the hERG, SCN5A, KCNQ1 and KCNQ1/MinK channels expressed in HEK-293 cells with IC_50_s of 6.0 µM, 7.5 µM, 3.5 µM and 3.7 µM respectively, and the KCND3/KChiP2 channels expressed in CHO cells with an IC_50_ of 10.6 µM. Analysis of celecoxib's effects on hERG channels suggested gating modification as the mechanism of drug action.

**Conclusions:**

The above channels play a significant role in drug-induced long QT syndrome (LQTS) and short QT syndrome (SQTS). Regulatory guidelines require that all new drugs under development be tested for effects on the hERG channel prior to first administration in humans. Our observations raise the question of celecoxib's potential to induce cardiac arrhythmias or other channel related adverse effects, and make a case for examining such possibilities.

## Introduction

Coxibs, selective inhibitors of cyclooxygenase-2 (COX-2), were developed to replace “traditional” non-steroidal anti-inflammatory drugs (NSAIDs) in the treatment of arthritis and acute pain. Four coxibs were marketed as NSAIDs with reduced gastrointestinal side effects. However, with high prevalence of serious cardiovascular complications among patients using Vioxx (rofecoxib), it was withdrawn in 2004, followed by removal of Bextra (valdecoxib) in 2005.

Celecoxib (Celebrex), one of the two coxibs remaining on the market (etoricoxib, or Arcoxia, is prescribed worldwide but not in the USA), also may cause an increased risk of serious and potentially fatal adverse cardiovascular thrombotic events, myocardial infarction, and stroke. The hazard ratio for the composite endpoint of cardiovascular complications is 3.4 for 400 mg celecoxib twice daily and 2.8 for 200 mg celecoxib twice daily compared to placebo [1].

Celecoxib targets cellular and enzymatic mechanisms other than cyclooxygenases, including voltage-activated Na^+^, K^+^ and Ca^2+^ channels [2]–[5]. We have shown that celecoxib can directly inhibit K^+^ channels in fruit flies, and K^+^ and Na^+^ channels in mammals, with strong effects on cardiomyocyte and neuronal function [6], [7]. We have also shown that celecoxib and SC-791 (a highly selective COX-2 inhibitor) can inhibit K_v_2.1 channels expressed in HEK-293 cells via modification of gating and channel block [8], [9]. In addition, it has been reported that celecoxib and its inactive analog, 2,5-dimethyl-celecoxib (DMC), but not rofecoxib, can acutely and reversibly up-regulate currents through K_v_7.5 (KCNQ5) cardiac channels, while inhibiting other currents [4]. Celecoxib can similarly inhibit K_v_1.5, K_v_7.1 and K_v_4.3 channels and alter action potential duration in mouse and guinea pig cardiomyocytes [10].

There are significant differences between the ionic basis of cardiac excitability in humans and that in animal models. In humans, drug effects on hERG, SCN5A (Na_v_1.5), KCNQ1/MinK and KCND3/KChiP2 (K_v_4.3) channels often lead to Long QT syndrome (LQTS) or Short QT syndrome (SQTS), including Torsade de Pointes (TdP) arrhythmias, fraught with ventricular fibrillation and ultimately death [11], [12]. In particular, hERG channel, the molecular basis of the rapidly activating K^+^ current (I_Kr_), is implicated in many life-threatening cardiac arrhythmias [13]–[18], and guidelines (ICH S7B) from regulatory agencies in the USA, European Union and Japan require that any new drug under development be tested for effects on the hERG current prior to first administration in humans [19].

The above observations on the widely prescribed celecoxib raise a question on its possible effects on hERG and other human channels. The main focus of this study was to examine the effect of celecoxib on hERG channels expressed in HEK-293 cells and the mechanism underlying this effect. In addition, we explored if celecoxib inhibited human SCN5A channels, examined concentration dependence of inhibition of human KCNQ1, KCNQ1/MinK and KCND3/KChiP2 channels [10], and studied the nature of inhibition of human KCNQ1/MinK channels.

## Results

### Inhibition of hERG channels

**Figure 1:**
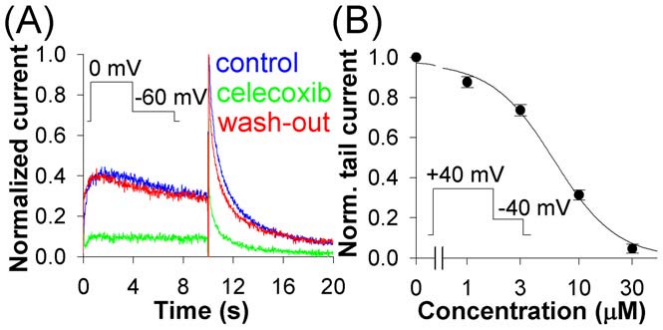
Effects of celecoxib on hERG. (*A*) The hERG current in control (blue), after application of 10 µM celecoxib (green) and after wash-out (red). The currents were elicited by a 10 s pulse to −60 mV following a 10 s pre-pulse to 0 mV (HP = −80 mV). (*B*) Dose-response relations for the peak tail currents evoked by a pulse to −40 mV after a 2 s pre-pulse to +40 mV; here and in the following figures the dose-response relations have been fitted to the Hill equation; number of cells varied between 3 and 11.

**Figure 2:**
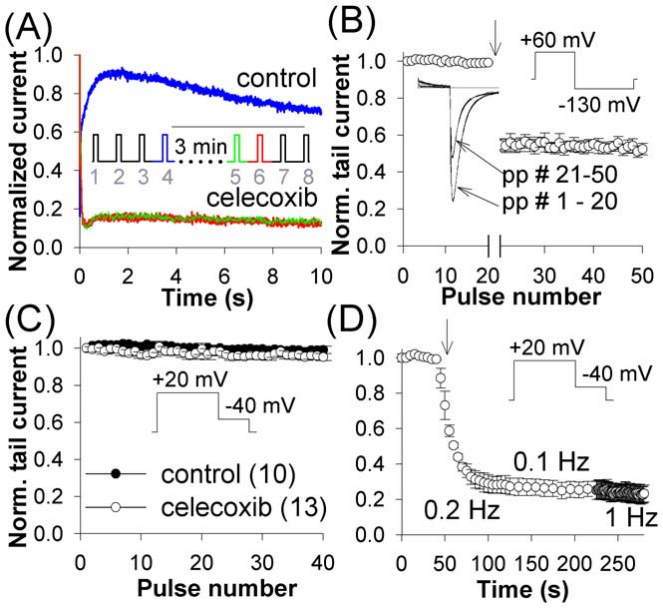
Onset of hERG inhibition. (*A*) Four 10 s pulses to 0 mV in control with 30 s inter-pulse intervals were followed by four pulses after 3 min exposure to 10 µM celecoxib. Data from 9 experiments were divided by maximum value of current in control in each cell and then averaged. Traces labeled ‘celecoxib’ overlap for pulses 5 (green) and 6 (red). (*B*) Celecoxib modulates closed hERG channels. Currents were elicited by 1 Hz pulse trains consisting of a 100 ms pre-pulse to +60 mV followed by a 150 ms pulse to −130 mV. After 20 pulses in control, the stimulation was ceased and 10 µM celecoxib was rapidly washed in (arrow). Stimulation was resumed after 3 min exposure to the drug while holding the cell at −80 mV; number of experiments, 4; inset shows an example of tail currents. (*C*) Inhibition of hERG was not use-dependent. Currents were evoked by 1 Hz pulse train consisting of a 400 ms pre-pulse to +20 mV followed by a 200 ms pulse to −40 mV and normalized to the magnitude of current during the first pulse in each sample; (n), number of experiments in this and the following figures. (*D*) Duration of the inter-pulse interval did not affect the extent of inhibition. Currents were elicited as in the panel *C*. After development of inhibition at 0.2 Hz in the presence of 10 µM celecoxib, the frequency of stimulation was first switched to 0.1 Hz for 10 pulses, and then to 1 Hz for 60 pulses; average of 3 cells.

The hERG channel is the most critical channel involved in genetic or drug-induced Torsade de Pointes (TdP) arrhythmias. Extracellular application of celecoxib caused rapid suppression of hERG1a channels (*Figures 1*, *2*). Celecoxib inhibited depolarization-induced current and the tail current to a similar extent (*Figure 1A*) suggesting a voltage-independent mechanism. 10 µM celecoxib inhibited the tail currents by 64% at −40 mV and 47% at −130 mV. IC_50_ was 6.0 µM (*Figure 1B*). The inhibition was reversible, with the time constant of 12.0±0.7 s for its onset (τ_on_) and 9.8±1.8 s for recovery from inhibition (_off_) at 10 µM celecoxib.

Examining effects of the drug on hERG during prolonged depolarizations can help distinguish between closed-channel block and a slow open-channel block. During the first instance of channel activation after application of an open-channel blocker, the blockade begins with relatively little inhibition. As more channels become blocked, the current decreases faster than in control. Because open-channel blockers usually dissociate from the channels held at hyperpolarizing potentials, such blockade is only partially relieved during inter-pulse intervals, thus resulting in consecutive current traces of gradually decreasing amplitude [20]–[22]. On the other hand, blockade of ion channels in their closed state(s) is often characterized by time- and voltage-dependent relief of inhibition during depolarization [23].

Experimental protocol consisted of eight identical voltage pulses (*Figure 2A*). The first four 10-s pulses from a holding potential (HP) of −80 mV to 0 mV applied in control to ensure stability of recordings were followed by a rapid wash-in of 10 µM celecoxib. Stimulation was resumed after 3 min exposure to celecoxib with the cell held at −80 mV. No difference was observed between currents for pulses 5 and 6 in the presence of the drug (green and red traces superimpose in *Figure 2A*-‘celecoxib’). This indicated that celecoxib either did not dissociate from hERG during inter-pulse interval (thus suggesting the unlikelihood of a slow open-channel block), or that open-channel block developed very fast. In addition, as there was no relief of inhibition during depolarizing pulse, the drug did not appear to block closed channels.

To examine the possibility of a rapid open-channel block, 1 Hz train of short depolarizations was used to elicit hERG tail currents at −130 mV (with 100 ms pre-pulse to +60 mV). After 20 pulses under control conditions, 10 µM celecoxib was added and allowed to equilibrate for 3 min with the cell held at −80 mV. By comparing the first tail current obtained in the presence of the drug with consecutive recordings and with current traces in control, it may be possible to detect a fast open-state block. Amplitude of the first tail in the presence of celecoxib was 55% of the last tail in control and did not decrease further with continued stimulation (*Figure 2B*). Similar observations were made with a 40 ms pre-pulse to +60 mV as well (data not shown). As the time constant for inactivation at +60 mV was 1.4±0.2 ms, only very rapid open-channel block with a comparable time constant could reach steady state level of inhibition during brief depolarizing pre-pulse without causing progressive reduction in tail currents. This experiment, however, cannot rule out a rapid inactivated-channel blockade (binding of a blocker to the pore of inactivated channel), which could have been established during the first occurrence of depolarization (100 or 40 ms pre-pulse to +60 mV). To further explore this possibility, use-dependence of hERG inhibition was tested at 1 Hz. No use-dependence of inhibition was found (*Figure 2C*).

A closed-channel blockade is characterized by use-dependent unblock [23]. This possibility was examined by stimulating the cells at different frequencies to see if inhibition was partially relieved at higher pulsing rates (*Figure 2D*). This protocol did not increase the tail current amplitude at 1 Hz, indicating the unlikelihood of closed-channel block.

### Modification of hERG1a gating

**Figure 3:**
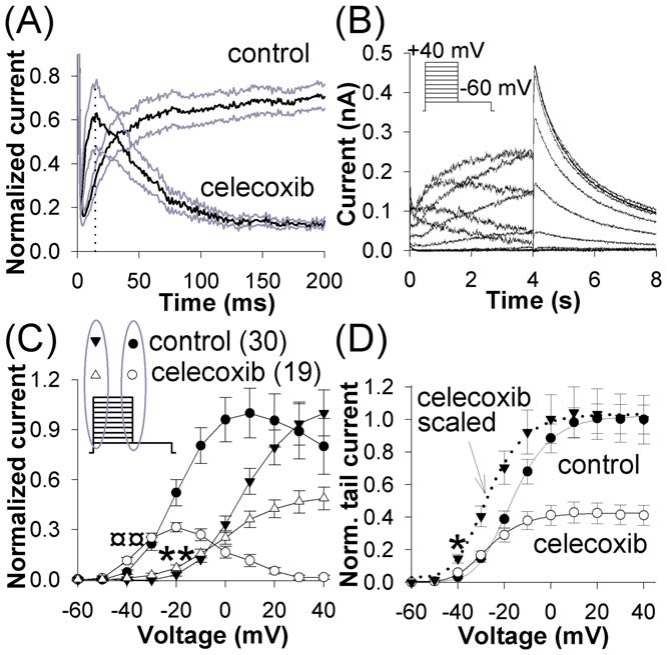
Modification of hERG gating by celecoxib. (*A*) Expanded first 200 ms from Fig. 2*A* shows a transient increase in hERG amplitude in the presence of celecoxib at 15 ms (vertical line) after the onset of pulse; gray traces indicate the range of SE. (*B*) Examples of hERG in control elicited by 4 s pulses to −60 mV after 4 s pre-pulses to voltages between −60 and +40 mV in 10 mV increments. (*C*) Normalized voltage-current relations for the sustained (end of a 4 s pre-pulse) and transient (maximal amplitude during the first 20 ms of pulse) components of hERG in control and at 10 µM celecoxib; currency signs mark statistical significance for circles, asterisks – for triangles. (*D*) Voltage-dependence of peak tail currents plotted against the pre-pulse voltage in control and at 10 µM celecoxib.

Closer examination of the initial 200 ms of recordings in *Figure 2A* revealed a “cross-over” of the current in control with that during the first few pulses in the presence of celecoxib (*Figure 3A*): the current in the presence of celecoxib transiently rose above that in control and peaked around 15 ms after the beginning of the voltage pulse (P<0.05, *t*-test).

Because transient increase in current on drug application is consistent with modification of channel gating but not with channel block, we explored celecoxib's effects on hERG kinetics. *Figure 3C,D* shows the effects of celecoxib on voltage-dependent properties of hERG after prolonged exposure. The current showed a threshold for activation at −50 mV, with inward rectification typical of hERG. Sustained current (current at the end of a 4 s pre-pulse) reached a maximum at +10 mV in control and at −20 mV in the presence of the drug. Importantly, current-voltage relations for the sustained currents demonstrated cross-over at −40 mV where the current in the presence of celecoxib was significantly larger than that in control (P<0.01, *t*-test, *Figure 3C*). Similarly to the transient increase in current during first instances of stimulation after drug application, there was a transient hERG increase even after prolonged drug exposure and repeated stimulation (at −20 mV, P<0.01, *t*-test, *Figure 3C*).

Deactivating tail currents, determined at −60 mV, displayed sigmoid voltage dependence and saturated at +10 mV. Celecoxib caused a hyperpolarizing shift in the half-activation potential (V^a^_½_) (determined using Boltzmann equation): from −16.2±1.2 mV in control to −27.6±1.5 mV at 10 µM celecoxib (*Figure 3D*). In addition, augmentation of the sustained currents in the presence of celecoxib resulted in the cross-over of I-V curves for the tail currents with significantly larger current (P<0.03) in the presence of celecoxib at −40 mV (*Figure 3D*).

**Figure 4:**
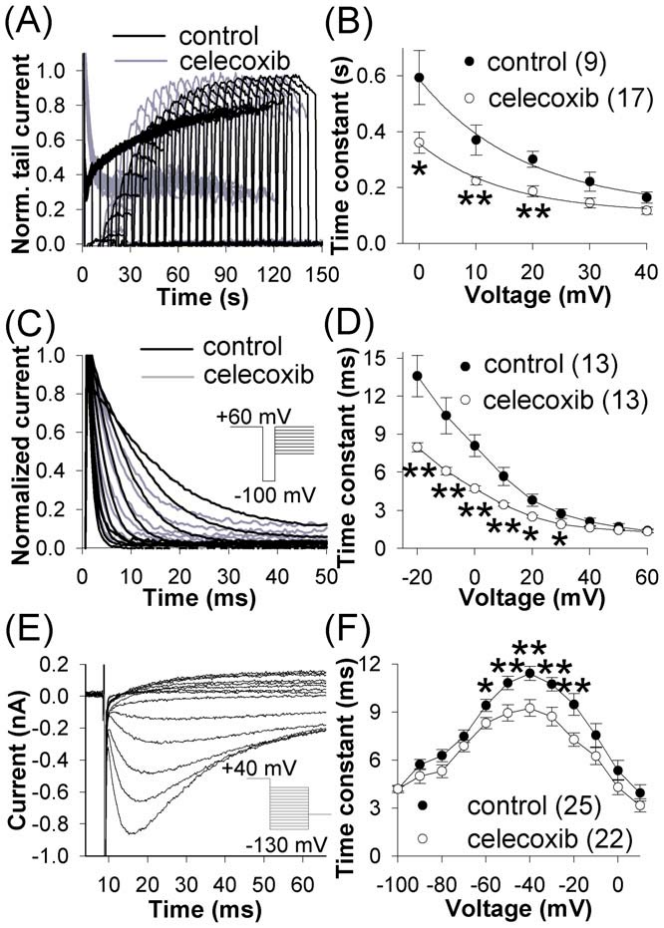
Celecoxib accelerated kinetics of activation, inactivation and re-activation of the hERG channels. (*A*) Examples of currents recorded during envelope-of-tails protocol to measure rates of activation at +20 mV in control and at 10 µM celecoxib. The protocol consisted of a variable duration (between 10 and 3,000 ms) pre-pulse to +20 mV followed by a test pulse to −60 mV. (*B*) Voltage dependence of the τ_act_. (*C*) Examples of inactivating currents in control and at 10 µM celecoxib. The protocol consisted of a 2 s first pre-pulse to +60 mV, a 10 ms second pre-pulse to −100 mV, and a test pulse between −20 and +60 mV in 10 mV increments. (*D*) Voltage dependence of the τ_inact_. (*E*) An example of re-activating current in control evoked by a 2 s pre-pulse to +40 mV followed by a 50 ms test pulse between −130 and +10 mV in 10 mV increments. (*F*) Voltage dependence of the τ_react_.

Activation time constants (τ_act_) were determined between 0 and +40 mV using the envelope of tail currents method (*Figure 4A*). The data points were fitted with a single exponential function to obtain τ_act_. Celecoxib significantly decreased τ_act_ between 0 and +20 mV (*Figure 4B*).

To obtain the inactivation time constants (τ_inact_), the time course of decay of large transient outward inactivating hERG current was fitted with a single exponential function. Celecoxib significantly reduced τ_inact_ between −20 and +30 mV (*Figure 4C,D*). Recovery of hERG from inactivation proceeds from inactivated to open state followed by deactivation. The time constants for re-activation (τ_react_) were determined by fitting the time course of re-activating currents (*Figure 4E*) with a single exponential function. The voltage dependence of recovery was bell-shaped and celecoxib decreased the values of τ_react_ between −60 and −20 mV (*Figure 4F*). Celecoxib did not affect deactivation kinetics of the hERG channels (data not shown).

### Inhibition of KCNQ1 and KCNQ1/MinK by celecoxib

While hERG channels are responsible for the largest proportion of drug-induced TdP arrhythmias, KCNQ1/MinK channels pass the slow cardiac delayed rectifier current I_Ks_ [24], [25] and also play a significant role in such arrhythmias. Loss of function mutations or pharmacological inhibition of KCNQ1 can result in LQTS forms 1 and 5 [24], [26]. Gain of function mutations in *KCNQ1* can cause SQTS and atrial fibrillation [27], [28].

**Figure 5:**
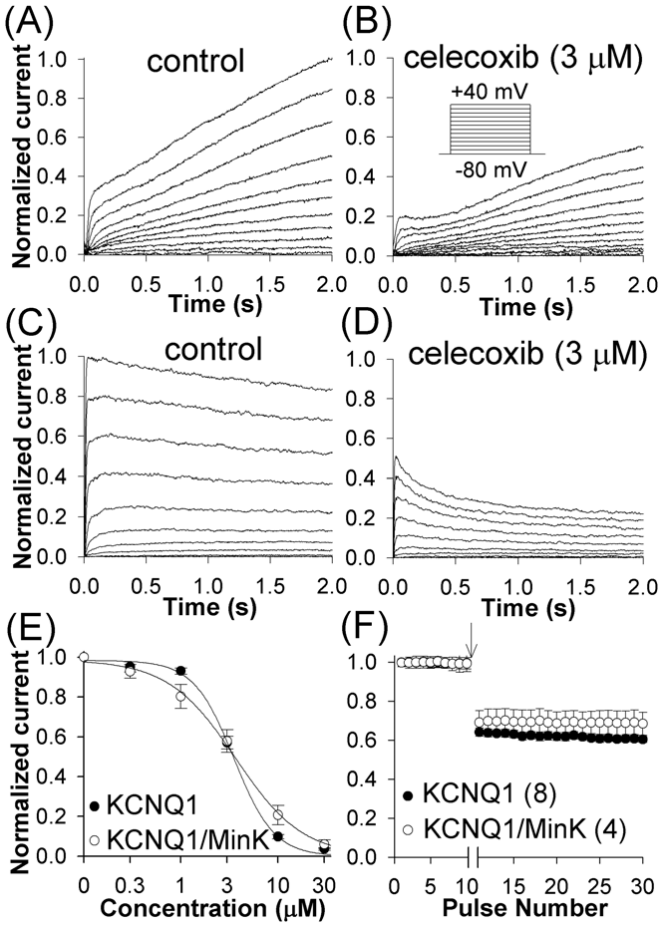
Effects of celecoxib on the KCNQ1/MinK and KCNQ1 channels. KCNQ1/MinK (*A, B*) and KCNQ1 (*C, D*) currents were elicited by voltage pulses between −80 mV and +40 mV in 10 mV increments (HP = −80 mV). (*E*) Dose-response relations for the KCNQ1/MinK current at the end of 2 s pulse and for the peak KCNQ1 current (at +40 mV); number of experiments varied between 3 and 18. (*F*) Celecoxib modulated closed channels; currents were elicited by 0.1 Hz train consisting of 1 s pulses to +40 mV. After 10 pulses in control, the stimulation was paused and 3 µM celecoxib rapidly washed in (arrow). Stimulation was resumed after 5 min exposure to celecoxib.

Celecoxib inhibited KCNQ1/MinK and KCNQ1 currents in concentration-dependent manner (*Figure 5*). The IC_50_ values were 3.7 µM for KCNQ1/MinK and 3.5 µM for KCNQ1 (*Figure 5E*).

Effects of celecoxib were reversible, with the τ_on_ of 42.3±1.4 s and the τ_off_ of 112.7±9.7 s for KCNQ1/MinK and τ_on_ of 37.1±2.4 s and τ_off_ of 114.1±14.8 s for KCNQ1. To determine if celecoxib exerted its effects on the closed channels, we examined the time course of the current after exposure of cells held at −80 mV, without electric stimulation, to 3 µM celecoxib. The protocol consisted of 0.1 Hz train of ten 1 s pulses to +40 mV under control conditions, a rapid application of 3 µM celecoxib with the cells held at −80 mV without stimulation, and resumption of stimulation after 5 min long exposure (*Figure 5F*). The amplitudes of the first pulses in the presence of the drug, after this stimulus-free exposure, were 69% and 64% of the controls for KCNQ1/MinK and KCNQ1, respectively and did not decrease with further stimulation, suggesting drug effect on closed channels. Celecoxib did not affect kinetics of activation or deactivation of these channels, nor did it alter the values of V^a^_½_.

### Inhibition of SCN5A

SCN5A channel is the major source of depolarization during action potential in human myocardium. Mutations in SCN5A can cause various cardiac disorders including LQTS and Brugada syndrome and the channel is the molecular target of antiarrhythmics, local anesthetics, and various off-mark medicines that may cause drug-induced LQTS [29], [30].

**Figure 6:**
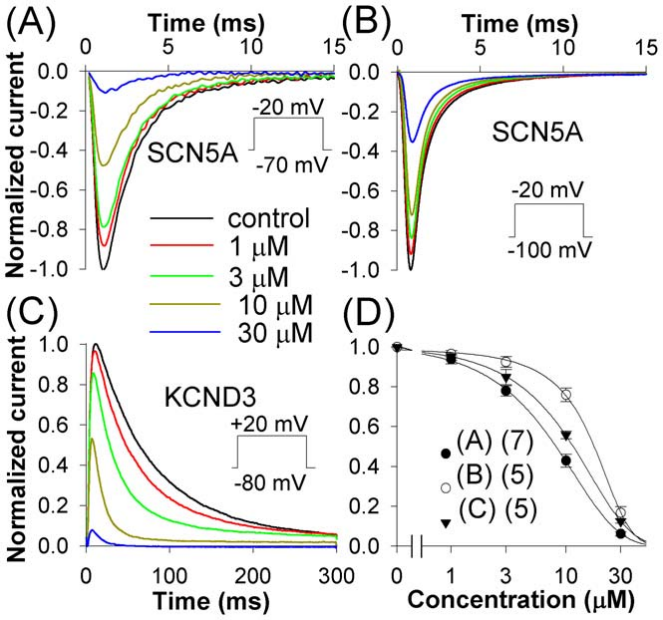
Effects of celecoxib on SCN5A and KCND3. (*A–B*) SCN5A currents were evoked by pulses to −20 mV from a HP of −70 mV (*A*) or −100 mV (*B*) in control and in the presence of different concentrations of celecoxib. (*C*) Inhibition of KCND3 channels. The currents were evoked by pulses to +20 mV from a HP of −80 mV. (*D*) Dose-response relations for inhibition of peak SCN5A and KCND3 currents; letters in the legend refer to the corresponding panels. *A–C*, legends are coded as in the panel *A*.

Although celecoxib was shown to inhibit sodium currents in rat dorsal root ganglion and retinal neurons [3], [7], the molecular bases of those currents are not known. In the present study we examined if celecoxib could inhibit human SCN5A. Celecoxib inhibited SCN5A currents evoked from the holding potentials (HP) of −70 and −100 mV with respective IC_50_s of 7.5 and 15.7 µM (*Figure 6A,B,D*).

### Inhibition of KCND3

The KCND3 channel constitutes an important component of cardiac transient outward potassium current I_to_ in human heart. Expression of KCND3 is altered in inherited and acquired cardiac pathologies such as Brugada syndrome or congestive heart failure [31], [32].

We examined effects of celecoxib on KCND3 channels stably expressed in the CHO cell line. Celecoxib inhibited the peak KCND3 current with an IC_50_ of 10.6 µM (*Figure 6C,D*).

## Discussion

Celecoxib has been shown to inhibit ion channels and induce arrhythmic beating in rat heart cells in cultures and *Drosophila* heart [6]. Here we show its inhibition of human ether-a-go-go related gene (hERG) channels, which are the most common substrate of drug-induced TdP arrhythmias in humans, and study the mechanism underlying this inhibition. In addition, the drug inhibited other human channels - SCN5A, KCNQ1/MinK and KCND3 - involved in TdP arrhythmias, in a concentration dependent manner.

The mechanism of hERG inhibition appeared to be modification of gating properties, rather than channel block. There was no indication of closed channel block. The current did not recover during prolonged depolarization (*Figure 2A*), inhibition was not affected by stimulation frequency (*Figure 2D*), and channel activation did not decelerate (*Figure 4*). Similarly, our data did not support open- or inactivated-channel block. Effects on amplitude and kinetics were completely set after 3 min exposure without stimulation. A rapid or a slow open-channel block was not likely as there was neither a progressive current decline upon repeated depolarization after exposure of closed channels to celecoxib, nor use-dependent decline (*Figure 2*). Ultra-fast open channel block with τ≤1.4 ms (the value of τ_inact_ at +60 mV) was unlikely because of the relatively slow onset of inhibition (τ_on_ of 12.0±0.7 s). Acceleration of recovery from inactivation (*Figure 4*) was inconsistent with inactivated-channel block, because the latter is associated with slowing of recovery due to need for the blocking molecule to dissociate from the channel pore before the channel can open again.

On the other hand, the data supported gating modification as the mechanism of effect on hERG and KCNQ1. The drug altered voltage-dependence of transitions between different states of the hERG channel with a facilitating effect as evidenced by changes in kinetics. It shifted V^a^_1/2_ towards less positive voltages by 11.4 mV, aiding activation and causing a transient increase in the current amplitude and cross-over of I–V curves in a way incompatible with channel block (*Figure 3*). It remained bound to closed, open, and inactivated hERG channels as evidenced by acceleration of the kinetics of activation, inactivation, and re-activation, respectively. Celecoxib's effects on KCNQ1 and KCNQ1/MinK were also compatible with gating modification. Although celecoxib did not affect channel kinetics except those of inactivation (*Figure 5*), its binding to closed channels was not consistent with open channel block.

Gating modification can accommodate a peculiar characteristic of changes in kinetics. As drug binding alters the gating rates by a fixed value of binding energy, the largest effects are expected at voltages that produce the smallest electrical driving force for the respective voltage-driven gating transitions, i.e. when the drug binding energy provides the largest relative contribution to the gating process. Indeed, celecoxib significantly decreased the τ_act_ of hERG below +30 mV, τ_inact_ below +40 mV, and τ_rec_ between −60 and −20 mV where the electrical driving forces for the respective transitions were relatively small.

Although celecoxib affects many channels, alters action potential duration in mouse and guinea pig cardiomyocytes [10], and induces arrhythmic beating in *Drosophila* heart and rat heart cells in culture, known differences between human and animal cardiac excitability [33] make it difficult to assess the human relevance of such findings. The human channels examined here, particularly hERG, are among the most prominent channels involved in drug-induced fatal TdP in humans.

Current regulations require any Investigational New Drug to be tested for effects on hERG before its administration in humans. At the same time, because some hERG inhibitors do not induce TdP and because a relatively small number of cardiovascular complications and fatalities due to celecoxib have been reported, it would be premature to conclude significant clinical implications from our data. On the other hand, the data do make a case for further exploring the possibility of such implications, as there are several factors that may influence clinical outcome of the usage of celecoxib, and while celecoxib may not induce arrhythmias in most patients, a small fraction of patients may be susceptible to arrhythmogenesis.

Adverse effects mediated through ion channels often occur at low, statistically inconspicuous, level in either pre-clinical trials or post-marketing usage. However, when a drug is widely used, such low-incidence effects may assume importance to public health. There are many cases of post-marketing withdrawal of drugs where cardiac arrhythmias arising from drug-induced channel dysfunction led to many deaths over the years before the seriousness of such effects was realized. For example, at one case per 100,000 patient-years of exposure, terfenadine (approved in 1985) led to 400 cases of serious injury or death due to cardiac arrhythmias before its withdrawal in 1998 [34]. Between 1990 and 2001, eight drugs were withdrawn due to cardiac arrhythmias arising from their use [35]. Many others have since been withdrawn, including Darvon (approved 1957; withdrawn 2010) [36].

Celecoxib inhibited the above channels at therapeutic plasma concentrations, which reach 2.8 µM (6.2 µM in women over 65) after a single dose of 200 mg and is often higher under many conditions such as moderate hepatic impairment or co-administration of drugs such as ketaconazole [37]. The drug is often used at dosages of 2×200 mg/day or 2×400 mg/day. Interpretations based on these values are complicated by two opposing factors. On one hand, 97% of the drug is bound to plasma proteins, leaving only 3% in free form [37]. On the other hand, the volume of distribution in humans is 455±166 Liters, making the total amount of drug present in the body close to 100 times that measured in plasma [37]. This indicates uneven tissue distribution with much higher concentration, and possibly stronger clinical implications, in some tissues than in others.

Moreover, significant physiological effects often occur at concentrations lower than IC_50_s for channel inhibition. For example, celecoxib inhibits Na^+^ channels in rat retinal neurons with IC_50_ of 5.2 µM, but inhibits neuronal firing mediated by these channels with IC_50_ of 0.76 µM [7]. This may occur because a partial inhibition of the Na^+^ current may prevent the membrane potential from reaching the threshold for firing. This would be similar to the significant risk of TdP known to develop from relatively small effects on hERG channels arising from genetic or pharmacological perturbations.

In addition to heart, the above channels express in many other tissues. For example, the hERG channels play an important role in smooth muscle contractility, pancreatic beta cell secretion, and neuronal excitability [38], and are involved in not only TdP but also other conditions including hyperinsulinemia, inefficient cortical processing, cognitive deficits and schizophrenia [38], [39]. It is premature to ascribe risk of cardiac arrhythmias or other adverse effects to celecoxib, but with more than one million prescriptions of Celebrex every month, the data make a case for exploring the implications of its inhibition of hERG and other human channels critical to cardiac rhythm and other physiological functions. Even when a drug is not withdrawn, being alert to its effects may save lives by taking precautions in specific patients who might be particularly vulnerable to arrhythmogenic, or other ion channel related, effects due to genetic or other factors.

On a general note, previous studies have established that ion channel inhibition is not a generic feature of coxibs, but rather that of celecoxib and some other structurally similar compounds not used in therapeutics [2], [4], [9]. Out of the four prescribed coxibs, effects on ion channels of only two of them, celecoxib and rofecoxib, have been investigated. Rofecoxib does not alter ion channel function [2], [4]. Of the remaining two, valdecoxib was removed from the market in 2005 and thus its possible action on ion channels is of no acute clinical importance. However, effects of etoricoxib on ion channels remain unknown and remain a possibly important issue to explore.

## Materials and Methods

Celecoxib was purified from commercial capsules as previously described, and was of >98% purity as determined by LC mass spectrometry and NMR spectroscopy^8^.

### Cell Culture

HEK-293 cells stably transfected with cDNA encoding hERG1a were kindly provided by Dr. G. Robertson [20]. The pCEP4-KCNQ1 and pCEP4-MinK vectors were kindly provided by Dr. Michael Sanguinetti [40]. To transiently express KCNQ1 and KCNQ1/MinK in HEK-293 cells, the sequences of human KCNQ1 and Mink were removed from pCEP4 and ligated into pcDNA 3.1 at the *Hin*d III and *Bam*H I sites for KCNQ1 and at the *Nhe* I and *Eco*R V sites for MinK. Sequence and directionality were verified by sequencing. The cells were grown in Dulbecco's Modified Essential Medium (Invitrogen, CA) at 37°C in 5% CO_2_.

One day before transfection, cells were plated on 35 mm Falcon culture dishes. Next day, 6 µL of FuGene 6 transfection reagent (Roche Molecular Biochemicals, IN), 2 µL of solution containing 1.5 µg pcDNA-KCNQ1 or 0.4 µg pcDNA-KCNQ1 plus 4 µg pcDNA-MinK, and 2 µL of solution containing 0.2 µg pEGFP-N2 were added to the Eppendorf tube containing 190 µL of DMEM, and gently shaken. After 30 min of incubation at room temperature, contents of the tube were added to the dish with HEK-293 cells. Recordings were performed 24–48 hours after transfection from cells displaying moderate green fluorescence.

Chinese hamster ovary (CHO) cells obtained from American Type Culture Collection (ATCC, P. O. Box 1549, Manassas, VA 20108) were stably transfected [41] with cDNAs encoding the human cardiac KCND3 channel and KChiP2 (Kv-Channel- Interacting Protein 2) with resistance to G418 and Zeocin (Invitrogen, CA) [41]. The cDNA encoding human SCN5A was stably transfected into HEK-293 cells as described previously [42]. Cells expressing these channels were grown in Ham's F-12 media supplemented with 10% foetal bovine serum at 37°C in 5% CO_2_.

### Patch-Clamp Recording and data analysis

Whole-cell current recordings and data analysis were performed using an Axopatch 200B amplifier and pClamp 9.2 software (Axon Instruments/Molecular Devices, CA). Patch electrodes were fabricated from thin-walled borosilicate glass (World Precision Instruments, Sarasota, FL). Electrodes had resistance of 1.0–3.5 MΩ. Access resistance was usually less than 10 MΩ and was compensated by 80% (no compensation was used for access resistance less than 3 MΩ).

For hERG1a and KCNQ1 (KCNQ1/MinK) recordings, electrodes were filled with the solution: 140.0 mM KCl, 5.4 mM NaCl, 2.0 mM MgCl_2_, 1.0 mM CaCl_2_, 11.0 mM EGTA, 10.0 mM HEPES (pH 7.2). The external solution contained 140.0 mM NaCl, 4.7 mM KCl, 1.2 mM MgCl_2_, 2.5 mM CaCl_2_, 10.0 mM HEPES, 11.0 mM glucose, pH 7.4 with NaOH. KCND3 and SCN5A channel recordings were performed as described previously [41], [42].

All experiments were performed at room temperature (20–22°C). All values are means ± S.E.M.; (*): P<0.05, (**): P<0.01 (paired two sample t-test for means or single factor ANOVA as indicated).
